# Efficacy of High‐Frequency Transcranial Magnetic Stimulation Therapy for Depression With Insomnia Symptoms: A Systematic Review and Meta‐Analysis Based on Randomized Controlled Trials

**DOI:** 10.1002/brb3.71808

**Published:** 2026-10-27

**Authors:** Wenqing Yang, Songbin Chen, Xiaotong Li, Junhui Chen, Li Li, Jin Yu

**Affiliations:** ^1^ Department of Rehabilitation Medicine Guangzhou First People Hospital Guangzhou Guangdong China; ^2^ Guangzhou University of Chinese Medicine Guangzhou Guangdong China; ^3^ Guangdong Food and Drug Vocational College Guangzhou Guangdong China

**Keywords:** comorbidity, depression, insomnia symptoms, left DLPFC, meta‐analysis, repetitive transcranial magnetic stimulation

## Abstract

**Background:**

This meta‐analysis evaluated the efficacy of high‐frequency repetitive transcranial magnetic stimulation (HF‐rTMS) targeting the left dorsolateral prefrontal cortex (DLPFC) in patients with comorbid depression and insomnia.

**Methods:**

This analysis incorporated 7 studies, involving 709 subjects (control: 342; repetitive transcranial magnetic stimulation [rTMS]: 367). All included studies employed left DLPFC‐targeted HF‐rTMS. Treatment effectiveness for comorbid depression and insomnia was assessed. Subgroup analyses were conducted to explore possible sources of heterogeneity.

**Results:**

HF‐rTMS significantly improved sleep quality (standardized mean difference [SMD] [95% confidence interval (CI)]: 0.97 [0.23 to 1.71], *p* = 0.01) and depressive symptoms (0.61 [0.38 to 0.84], *p* < 0.00001). There was a non‐significant trend toward reduced sleep latency (0.50 [−0.17 to 1.18], *p* = 0.14). No significant difference in adverse reactions was observed (risk ratio [95% CI]: 1.09 [0.80 to 1.48], *p* = 0.57). Substantial heterogeneity was identified for the Pittsburgh Sleep Quality Index outcomes (*I* ^2^ = 91%). Subgroup analyses indicated that this heterogeneity was attributed to variations in stimulation frequency and baseline antidepressant use.

**Conclusion:**

Very‑low to low certainty evidence suggests that left DLPFC‑targeted HF‑rTMS may improve sleep quality and depressive symptoms in patients with depression and insomnia symptoms, with a favorable safety profile. However, due to substantial heterogeneity and methodological limitations, these findings should be considered exploratory and warrant confirmation by future high‑quality trials.

## Graphical abstract

It summarizes the key findings: improves sleep quality (SMD0.97) and depressive symptoms(SMD0.61) in depression‐related insomia

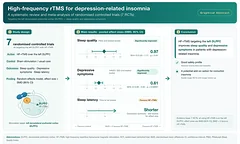

## 1 Introduction

Depression represents a prevalent mental condition with high rates of incidence, recurrence, and disability. It severely impairs psychosocial function and quality of life (Malhi and Mann 2018). In China, depression is forecast to emerge as the second principal cause of disability (Lu et al. 2021). Evidence suggests that depression and insomnia often occur together, likely due to bidirectional interactions involving the hypothalamic‐pituitary‐adrenal axis, the monoamine hypothesis, circadian rhythm disorders, and mood disorders. These pathophysiological interactions may lead to reduced prefrontal cortex excitability, thereby exacerbating both conditions (Buysse et al. 2008; Gold and Chrousos 2002; Hirschfeld 2000; McClung 2013).

Although antidepressants are effective, their use can be limited by side effects, non‐response, relapse, and withdrawal symptoms (Fava et al. 2015; Montejo et al. 2015). Indeed, approximately two‐thirds of patients may require long‐term medication or fail to respond adequately to initial therapy (Chen et al. 2023; Henssler et al. 2024; Kamp et al. 2024). Consequently, developing effective nonpharmacological alternative treatments for depression with comorbid insomnia is urgently required.

Repetitive transcranial magnetic stimulation (rTMS) is a noninvasive neuromodulatory technique increasingly used in depressive disorders (Joseph et al. 2024; Pu et al. 2023). High‐frequency rTMS (HF‐rTMS) over the left dorsolateral prefrontal cortex (DLPFC) enhances cortical excitability and has shown promise in alleviating both depressive and sleep symptoms (Teng et al. 2017). However, prior meta‐analyses have primarily focused on mood outcomes, treating insomnia as a secondary measure rather than a prespecified therapeutic target (Liu et al. 2025). Studies examining rTMS for sleep disturbances have largely enrolled patients with primary insomnia and used heterogeneous targets (e.g., parietal or motor cortex) and frequency protocols. This substantially limits the generalizability of their findings to the depressive‐insomnia comorbidity (Feng et al. 2025; Jesús et al. 2025; Khosravi and Khosravi 2025; Xia et al. 2025). Moreover, no existing review has systematically distinguished the sleep‐promoting effects of HF‐rTMS from its mood‐improving effects, nor explored dose‐response relationships between specific stimulation parameters (e.g., total pulses, frequency, intensity) and comorbid outcomes (Carè et al. 2024; Zhao et al. 2024). This knowledge gap critically undermines the translational value of current evidence for guiding clinical decision‐making in this specific patient population.

These alternative technologies are beyond the scope of this review, and their effects on sleep‐related circuits remain unexplored. Neuroimaging has identified prefrontal–limbic–thalamic dysfunction as a shared biomarker in depression‐insomnia comorbidity, supporting left DLPFC‐rTMS as a rational target (Jin et al. 2025; Varone et al. 2025). Yet current protocols lack EEG‐based or symptom‐driven stratification, leaving personalization for comorbid patients clinically insufficient. Critically, it remains unclear whether HF‐rTMS improves sleep independently of mood improvement or only as a secondary effect—a distinction essential for optimizing target selection and combination strategies. Recent advances in non‐invasive brain stimulation (e.g., accelerated TMS, TBS, transcranial electrical stimulation, and resting‐state fMRI‐guided targeting) have improved efficacy in treatment‐resistant depression but fall outside the scope of this review (He et al. 2025; Kan et al. 2023; Lefaucheur et al. 2020; Wei et al. 2025).

To address these gaps, we conducted a systematic review and meta‐analysis of randomized controlled trials (RCTs) to: (i) evaluate the independent treatment effects of left DLPFC‐targeted HF‐rTMS on depression with insomnia symptoms, (ii) identify modulators of treatment efficacy through subgroup analyses, and (iii) provide effect‐size estimates to guide future trial design, ultimately offering higher‐level evidence to inform personalized neuromodulation in this understudied population.

## 2 Methods

### 2.1 Study Design and Registration

The present meta‐analysis complied with the Preferred Reporting Items for Systematic Reviews and Meta‐Analyses statement and was registered with the International Prospective Register of Systematic Reviews on March 20, 2026 (registration ID: CRD1112579).

### 2.2 Search Strategy

The Cochrane Library, Web of Science, Embase, CNKI, PubMed, and WanFang were searched up to July 15, 2025. The search strategy combined keywords and medical subject headings: (“sleep initiation and maintenance disorders” OR “insomnia”) AND (“depressive disorder” OR “depression”) AND (“transcranial magnetic stimulation” OR “rTMS”).

### 2.3 Eligibility Criteria

Inclusion criteria: (i) Subjects fulfilling standardized diagnostic criteria for depression (Chinese Classification of Mental Disorders, Third Edition [CCMD‐3], International Classification of Diseases, 10th Revision [ICD‐10], and Diagnostic and Statistical Manual of Mental Disorders, Fourth Edition [DSM‐IV]). Patients with depressive disorders of various etiologies (major depressive disorder [MDD], post‐stroke depression [PSD], substance‐induced) who also presented with insomnia symptoms were included, as they share common prefrontal‐limbic dysregulation mechanisms that provide a neurobiological rationale for pooling; (ii) age ≥ 17 years; (iii) comorbid insomnia, defined as a Pittsburgh Sleep Quality Index (PSQI) global score > 5 (used as a screening criterion for clinically significant insomnia symptoms rather than a formal insomnia diagnosis) or a Hamilton Depression Rating Scale (HAMD) insomnia item score > 2; (iv) RCT design (parallel or crossover) with full text available in English or Chinese; (v) Intervention involving left DLPFC‐targeted HF‐rTMS (stimulation frequency ≥ 5 Hz); (vi) Baseline antidepressant use was permitted but not required.

Exclusion criteria: (i) non‐randomized studies, animal studies, reviews, or conference abstracts; (ii) trials involving subjects with bipolar disorder or schizophrenia; (iii) secondary insomnia due to other conditions; (iv) interventions were low‐frequency rTMS (≤ 3 Hz) or targeting non‐DLPFC regions; (v) combination with other physical therapies; (vi) lack of a sham‐control group; (vii) insufficient description of sham stimulation parameters; (viii) unreported or incomplete outcome data; (ix) studies from which it was infeasible to extract or calculate effect sizes.

### 2.4 Outcome Measures

Primary outcomes incorporated: (i) sleep quality, quantified by PSQI total score, and (ii) depressive symptoms, assessed by HAMD‐17 score. Both were analyzed as standardized mean differences (SMDs).

Secondary outcomes comprised clinical remission rate (proportion of subjects achieving a PSQI score < 5 or an HAMD insomnia item score ≤ 1), incidence of adverse events (AEs), and changes in sleep architecture parameters. TMS‐related AEs were defined as any unintended, non‐therapeutic physiological or psychological reactions occurring during or after stimulation that may cause discomfort or harm but resolve spontaneously without specific medical intervention. Common manifestations include: (i) head discomfort and headache, resulting from scalp muscle contraction or vasodilation and resembling tension‐type headache; (ii) local pain or sensory abnormalities, such as tingling, numbness, burning, or facial muscle twitching at the stimulation site; and (iii) auditory discomfort, including tinnitus or ear fullness due to device noise when earplugs were not used.

### 2.5 Data Extraction

To ensure the accuracy and consistency of data extraction, a standardized form was developed. YWQ and CSB extracted the data independently, followed by cross‐verification. Disagreements were settled via consultation with LXT. The extracted data encompassed: (i) publication details: publication year, first author, and the nation where the research was performed; (ii) subject information: effective sample size per group, sex, age, nationality, and illness duration; (iii) rTMS protocols: intensity, frequency, stimulation site, total number of sessions, inter‐train interval, and total pulse count; (iv) baseline antidepressant use; (v) number and type of adverse reactions; (vi) primary endpoints: mean values and standard deviations for depression and insomnia scale scores (HAMD, PSQI, sleep latency [SL]) before and after intervention.

### 2.6 Quality Evaluation

We used the revised Cochrane Risk‐of‐Bias 2.0 (RoB 2) tool for parallel‐group RCTs. This tool was employed to evaluate RoB in the following domains: random sequence generation (selection bias), blinding of participants and personnel (performance bias), allocation concealment (selection bias), blinding of outcome assessment (detection bias), selective reporting (reporting bias), and other biases. Each domain was categorized as having high, low, or unclear RoB.

### 2.7 Statistical Analysis

All statistical analyses were performed using review manager (v5.3). For continuous outcomes (changes in depression, insomnia, and SL scores), pooled SMDs with 95% confidence intervals (CIs) were computed to accommodate different measurement scales. Because lower PSQI and HAMD scores indicate greater improvement, endpoint values or change‐from‐baseline scores (baseline minus endpoint) were used such that a positive SMD consistently favored the intervention group. For studies reporting both, we preferentially used change‐from‐baseline scores; when only endpoint values were available, we used them. All scales (PSQI, HAMD) were scored such that lower values indicated better outcomes; we used endpoint values or change scores (baseline minus endpoint) consistently so that a positive SMD always favored the intervention group. Variance was calculated from reported CIs, t‐values, or p‐values according to Cochrane Handbook recommendations. For dichotomous outcomes (e.g., AEs), pooled risk ratios (RRs) with 95% CIs were derived, as RRs offer greater clinical interpretability.

For all meta‐analyses, a random‐effects model (DerSimonian‐Laird) was applied to account for anticipated methodological and clinical heterogeneity. Statistical heterogeneity across the eligible trials was evaluated via the Chi^2^ test (with p‐value < 0.10 indicating potential heterogeneity) and the I^2^ statistic, categorized as: 75%–100% (considerable); 50%–90% (moderate to substantial); 30%–60% (low to moderate); and 0%–40% (inconsequential). For primary outcomes with substantial heterogeneity (*I* ^2^ > 50%), subgroup analyses stratified by diagnostic criteria (e.g., CCMD‐3 vs. DSM‐IV vs. ICD‐10), stimulation frequency (e.g., 10 vs. 15 Hz), and baseline antidepressant use were performed to identify potential sources of heterogeneity.

### 2.8 Certainty of Evidence (Grading of Recommendations, Assessment, Development, and Evaluations [GRADE] Assessment)

We assessed the certainty of evidence for each outcome using the GRADE framework. Evidence was rated as high, moderate, low, or very low across five domains: risk of bias, inconsistency, indirectness, imprecision, and publication bias. Two independent reviewers performed the assessments using GRADEpro GDT; discrepancies were resolved by consensus. A summary table of findings was constructed for the primary outcomes (PSQI, HAMD‐17) and secondary outcomes (SL, AEs).

## 3 Results

### 3.1 Study Screening and Characteristics

Two articles were excluded after full‐text review: one because it was a master's thesis, and the other because it did not meet the diagnostic criteria. Ultimately, seven RCTs using left DLPFC‐targeted HF‐rTMS for comorbid depression and insomnia were included, involving 709 subjects (control: 342; rTMS: 367) (Chen et al. 2020; Lin et al. 2019; Lu et al. 2022; Pu et al. 2023; Rosenquist et al. 2013; Triggs et al. 2010; Xu et al. 2024). Among these, Studies 14 and 17 focused specifically on MDD. Figure 1 illustrates the screening process. The high number of non‐retrieved reports (131/140) was primarily due to: (a) conference abstracts without subsequent full‐text publication; (b) articles not indexed in our accessible library databases despite repeated retrieval attempts. Table S2 presents the results of literature search, and Table S3 provides the PRISMA 2020 checklist. Table S4 contains our raw data; all data is derived from this table. Table 1, Table 2 summarizes the demographic characteristics, intervention strategies, and efficacy evaluation metrics of the subjects. Treatment duration ranged from two to 12 weeks across studies, and assessment tools included the HAMD, PSQI, and SL scores.

**FIGURE 1:**
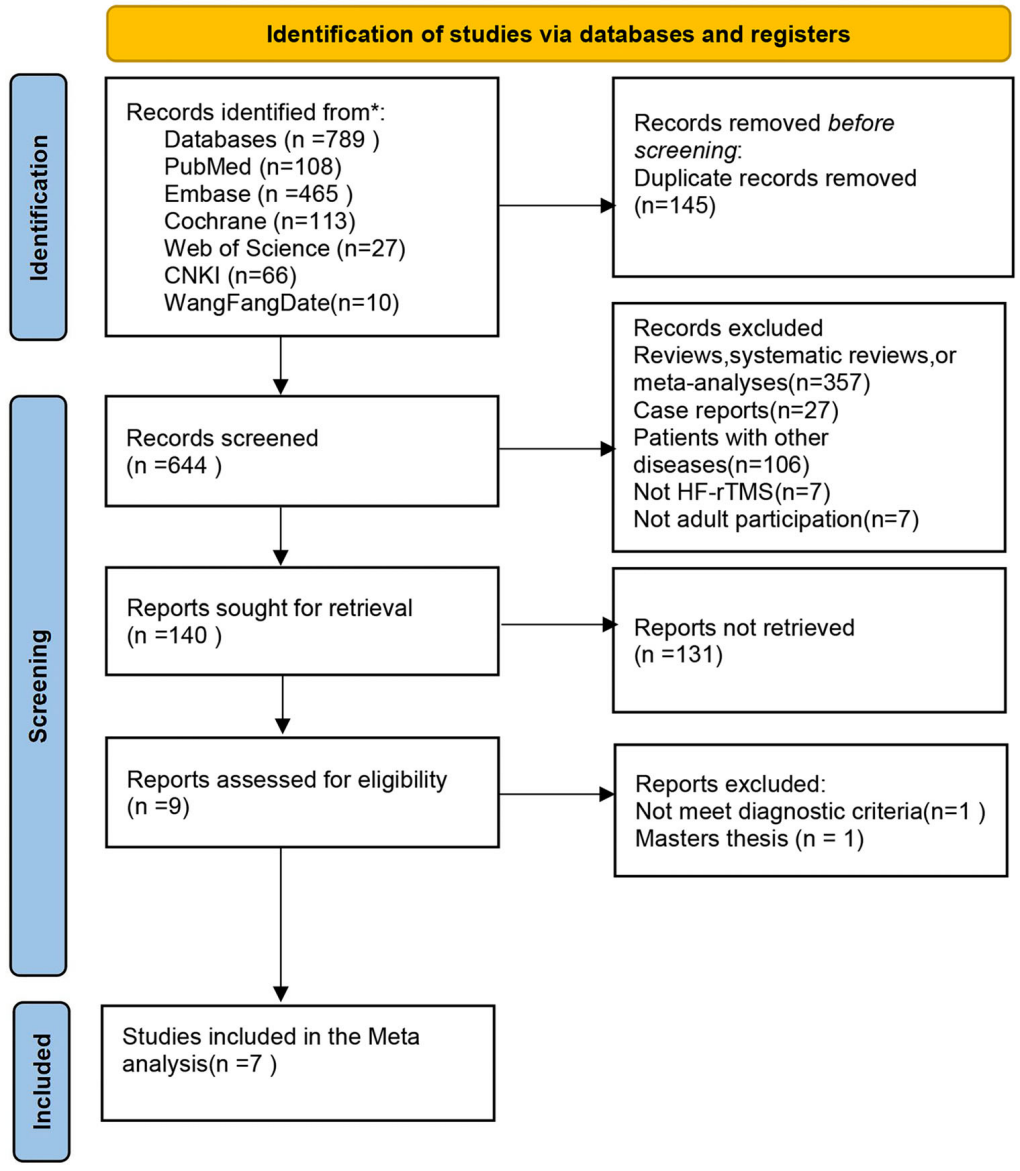
PRISMA flow diagram of the study selection process. The diagram outlines the study identification, screening, and inclusion process, resulting in 7 studies included in the final meta‐analysis.

**TABLE 1:** Demographic characteristics of included studies.

Study ID	Country	Sample size (Total)	Group size (T/C)	Age (years)	Gender (male.n%)	Diagnostic criteria	Remark
Puzhengping2023	China	82	*T* = 42 *C* = 40	T:35.24 ± 5.12 C:33.97 ± 4.74	T:19(45.2%) C:22(55%)	International Classification of Diseases (Tenth Edition) (ICD‐10), (HAMD‐17) score range: 7–23, (PSQI) score > 7	Major depressive disorder
WilliamJ 2010	USA	25	*T* = 18 *C* = 7	T:46.7 ± 15.3 C:41.9 ± 14.1	T:4(22.2%) C:5 (71.4)	Major depressive disorder、 DSM IV、HAMD‐24 score ≥ 18、and HAMD item number 1 score ≥ 3	Major depressive disorder
Peter B2013	USA	301	*T* = 155 *C* = 146	T:47.9 ± 11.0 C:48.7 ± 10.6	T:69(44.5%) C:74(49.3%)	Major depressive disorder、DSM‐IV	Major depressive disorder
JianLin2019	China	80	*T* = 40 *C* = 40	T:33.95 ± 7.13 C:34.5 ± 8.54	T:40(100%) C:40(100%)	Drug dependence 、DSM‐IV、PSQI	
Xuxiafei2024	China	48	*T* = 25 *C* = 23	T:60.62 ± 11.01 C:59.6 ± 10.54	T:14(56%) C:11(47.8)	2016 Chinese expert consensus on clinical practice for PSD、ICD‐10、 HAMD‐17 scale ≥ 7、 PSQI score ≥ 8	
Chenliping2020	China	63	T:32 C:31	T:64.06 ± 6.82 C:65.16 ± 9.18	T:23(71.8%) C:21(67.7)	CCMD‐3、HAMD‐17 score ≥ 8 、PSQI score ≥ 8	
Lule2022	China	110	T:55 C:55	T:33.85 ± 8.16 C: 35.04 ± 9.22	T:22(40.0%) C:27(9.0%)	CCMD、PSQI score > 5	

author, year, country, sample size, age, sex, diagnostic criteria, and clinical subtype.

**TABLE 2:** Clinical characteristics of included studies.

Study ID	Baseline medication	Intervention parameters	HAMD (M±SD) baseline, end point	PSQI (M±SD) baseline, end point,	Comorbidities	Follow‐up week	Outcomes
Puzhengping2023	Agomelatine25‐70 mg/d、8week	T:10HZ、Left DLPFC120%MT、25 trains of 8s、26‐s train interval 5times/week、20sessions C:Sham coil perpendicular to scalp, same regimen	T0:22.82 ± 6.39 T4:8.57 ± 3.25 T8:4.43 ± 31.13 C0:21.45 ± 6.56 C4:11.13 ± 3.74 C8:6.39 ± 1.27	T0:14.62 ± 3.84 T4:7.06 ± 2.87 T8:4.38 ± 1.24 C0:14.24 ± 3.67 C4:8.68 ± 2.11 C8:6.72 ± 1.53	NA	8	1.3.4
WilliamJ 2010	NA	T:5HZ、l eft‐DLPFC、100%MT、50 trains × 40 stimuli, 8 s/train, 2000 total、2 week C:A Magstim placebo coil system (Magstim) same regimen	T0:28.2 ± 6.0 T4:18.2 ± 9.8 T12:16.3 ± 11.5 C0:27.7 ± 3.5 C4:26.0 ± 9.2 C12:25.8 ± 7.4	NA	NA	12	2.4
Peter B2013	Lorazepam 2 mg/day 、14 day	T:L‐DLPFC、10HZ、120%MT、4 s on / 26 s off cycle、3000 pulses、5d/week/6 week C:NA	T0:30.7 ± 3.9 C0:30.6 ± 4.3	NA	NA	6	2.4
JianLin2019	NA	T:10HZ、100%MT、L‐DLPFC、5‐s on, 10‐s off for 10 min, 2000 pulses、5d/week/6week C:L‐DLPFC, sham coil perpendicular to scalp, same regimen	NA	T0:10.95 ± 1.34 T6:8.03 ± 0.83 C0:12.78 ± 1.25 C6:12.68 ± 1.19	NA	6	3.4
Xuxiafei2024	Enteric‐coated paroxetine20‐50 mg、Clonazepam1‐3 mg、5 times	T:10 Hz、 intensity of 90%、 L‐ DLPFC(F3)、4 s active, 26 s pause, 3000 pulses、5d/week/2week C:Sham coil perpendicular to scalp, same regimen	T0:28.72 ± 7.87 T2:11.40 ± 4.21 T6:9.28 ± 3.77 C0:29.87 ± 5.82 C2:16.70 ± 4.24 C6:11.83 ± 2.92	T0:16.96 ± 2.59 T2:10.88 ± 2.42 T6:9.44 ± 2.00 C0:16.83 ± 1.87 C2:14.17 ± 1.97 C6:13.57 ± 3.36	6–24 months post‐stroke	6	1.3.4
Chenliping2020	Escitalopram oxalate 10 ∼ 20 mg/day	T:10HZ、90%MT、left DLPFC、 5‐s off for 1 min, 20 stimulationsnds、5d/week、2 week C:Sham coil perpendicular to scalp, same regimen	T0:20.25 ± 2.24 T4:8.35 ± 4.58 C0:20.28 ± 2.13 C4:11.06 ± 3.81	T0:12.06 ± 5.85 T4:6.16 ± 4.17 C0:11.52 ± 5.51 C4:6.85 ± 4.20	>2 months post‐stroke	4	1.3.4
Lule2022	Agoematin tablets 25–50 mg/day、1month	T:15HZ、L‐DLPFC、80%–85%RMT、5 s on, 10 s off, 10 min total, 5d/week、12 week C:Sham coil perpendicular to scalp, same regimen	T0:28.23 ± 4.41 T12:8.61 ± 0.73 C0:28.05 ± 4.57 C12:10.54 ± 1.26	T0:15.11 ± 2.76 T12:6.10 ± 2.14 C0:15.29 ± 2.84 C12:6.97 ± 2.03	NA	12	1.3.4

TMS parameters: frequency, intensity, site, pulses, course duration, and follow‐up and outcome variables: PSQI and HAMA baseline and endpoint values

### 3.2 Study Quality

Overall, the RoB across the eligible RCTs was moderate to low. All studies employed randomization for subject allocation. Every article reported the methods for generating random sequences; however, none described allocation concealment. Three studies provided detailed double‐blind procedures, and one evaluated incomplete outcome data (loss to follow‐up bias) to avoid detection bias. Details are illustrated in Figure 2. Following the exclusion of the two studies rated as having a high risk of bias based on the ROB 2.0 tool, the meta‐analysis yielded a pooled SMD of 1.02 (95% CI: −0.07 to 2.11, *p* = 0.07). Heterogeneity remained considerable (*I* ^2^ = 94%, *p* < 0.00001; Figure S2).″

**FIGURE 2:**
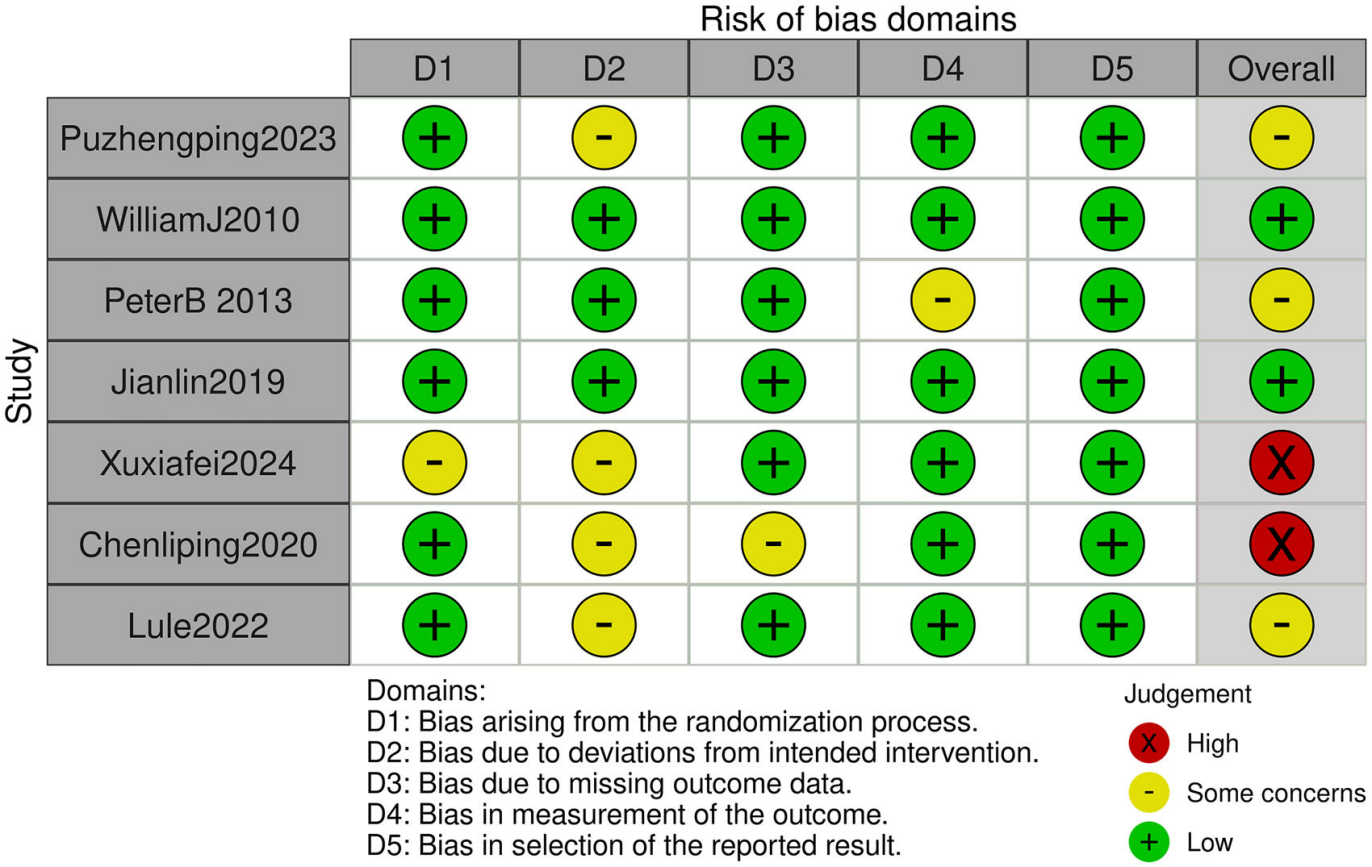
Methodological quality assessment of the 7 included studies using the Cochrane Risk of Bias 2.0 (RoB2.0) tool across five domains. D1 (bias arising from the randomization process): 5 studies rated as low risk (green), 2 studies (Xuxiafei 2024, Chenliping 2020) with some concerns (yellow). D2 (bias due to deviations from intended interventions): 3 studies rated as low risk, 4 studies with some concerns (yellow). D3 (bias due to missing outcome data): all 7 studies rated as low risk (green). D4 (bias in measurement of the outcome): 6 studies rated as low risk, 1 study (PeterB 2013) with some concerns (yellow). D5 (bias in selection of the reported result): all 7 studies rated as low risk (green).

### 3.3 Primary and Secondary Outcomes

#### 3.3.1 Primary Outcomes

The pooled analysis revealed that HF‐rTMS significantly improved sleep quality relative to the control group (SMD = 0.97, 95% CI: 0.23 to 1.71, *p* = 0.01; Figure 3), albeit with substantial heterogeneity (*I* ^2^ = 91%, *p* < 0.00001).

**FIGURE 3:**
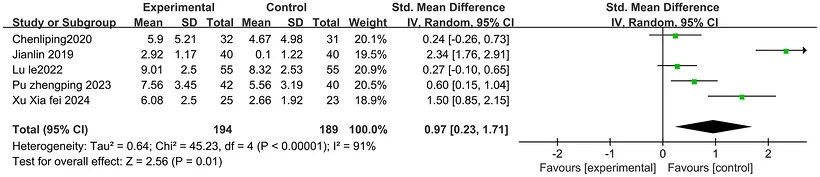
Forest plot of PSQI scores. Effect sizes are standardized mean differences (SMDs) with 95% confidence intervals (CIs) using a random‐effects model. The pooled SMD was 0.97 (95% CI: 0.23 to 1.71, *p* = 0.01). Heterogeneity: *I* ^2^ = 91%, *p* < 0.00001.

HF‐rTMS also significantly reduced depressive symptoms (SMD = 0.61, 95% CI: 0.38 to 0.84, *p* < 0.00001; Figure 4) with negligible heterogeneity (*I* ^2^ = 0%, *p* = 0.83). Additionally, we conducted leave‐one‐out sensitivity analyses for the two outcome measures, PSQI and HAMD. After sequentially excluding individual studies, the pooled effect sizes remained robust. No single study was found to have a driving effect on the overall outcomes.

**FIGURE 4:**
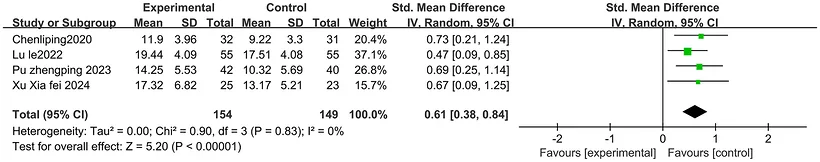
Forest plot of HAMD scores. Effect sizes are standardized mean differences (SMDs) with 95% confidence intervals (CIs) using a random‐effects model. The pooled SMD was 0.61 (95% CI: 0.38 to 0.84, *p* = 0.83). Heterogeneity: *I* ^2^ = 0%, *p* < 0.00001.

Additionally, the pooled analysis of SL showed a non‐significant trend toward reduction (SMD = 0.50, 95% CI: −0.17 to 1.18, *p* = 0.14; Figure 5). This analysis included only two studies with 145 participants, and substantial heterogeneity was detected (*I* ^2^ = 75%, *p* = 0.04). Given that no subgroup analysis was implemented owing to the small number of eligible studies, this result should be interpreted as exploratory.

**FIGURE 5:**
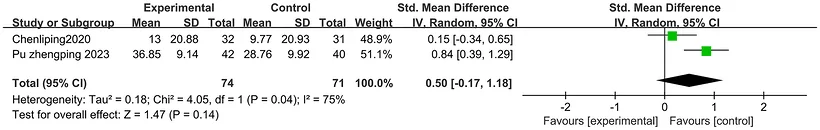
Forest plot of SL scores. Effect sizes are standardized mean differences (SMDs) with 95% confidence intervals (CIs) using a random‐effects model. The pooled SMD was 0.50 (95% CI: 0.17 to 1.18, *p* = 0.14). Heterogeneity: *I* ^2^ = 75%, *p* = 0.04. This analysis included only two studies (*n* = 145 participants); therefore, this finding is exploratory and should be interpreted with caution.

#### 3.3.2 Secondary Outcomes

All seven RCTs reported on adverse reactions through active monitoring, with most AEs being mild and no serious adverse events reported. The pooled analysis showed no significant difference in the frequency of AEs between the HF‐rTMS and control groups (RR = 1.09, 95% CI: 0.80 to 1.48, *p* = 0.57; Figure 6). Nevertheless, the lack of a significant difference in overall AE rates should not be interpreted as evidence of safety equivalence. Given the limited number of trials and small total sample size, confidence in this safety estimate is low, and larger, longer‐term studies are needed to adequately characterize the safety profile of HF‐rTMS in this population.

**FIGURE 6:**
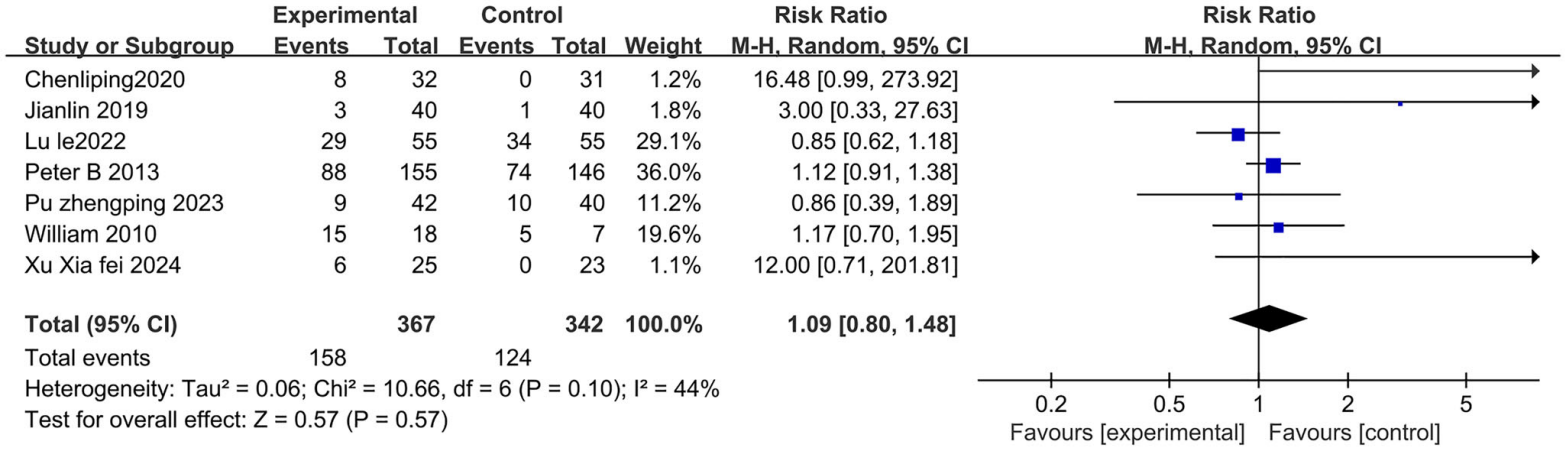
Forest plot of risk ratios for adverse events. Effect sizes are presented as risk ratios (RRs) with 95% confidence intervals (CIs), estimated using a random‑effects model. The pooled RR was 1.09 (95% CI: 0.80 to 1.48, *p* = 0.57). Heterogeneity was moderate (*I* ^2^ = 44%, *p* = 0.10).

### 3.4 Subgroup Analysis

To explore the substantial heterogeneity observed in the PSQI outcomes (*I* ^2^ = 91%, *p* < 0.00001), subgroup analyses were conducted (Figure 7). Analyses based on diagnostic criteria (ICD‐10, DSM‐IV, and CCMD‐3) showed consistent effect directions and low within‐subgroup heterogeneity, indicating that diagnostic criteria were not a major source of heterogeneity. The baseline antidepressant use subgroup included all participants who received antidepressants consistently throughout the study, while the no‐baseline antidepressant use subgroup consisted of those who neither requested nor reported baseline antidepressant use. Potential sources of heterogeneity were identified through analyses of stimulus frequency and baseline medication status. The effect size was significant and homogeneous in the 10 Hz subgroup (SMD = 1.15, 95% CI: 0.14 to 2.07). Only one study was available for the 15 Hz subgroup (SMD = 0.47, 95% CI: 0.09 to 0.85), precluding further heterogeneity analysis. Similarly, substantial heterogeneity was present among subjects using baseline medications, whereas those not using medications showed a homogeneous and significant effect size. Thus, differences in stimulation frequency and concurrent baseline antidepressant use may have partially explained the observed heterogeneity. Additionally, subgroup analysis of population characteristics revealed significant differences among subgroups (*p* < 0.0001), indicating variation in treatment effects across patient groups. However, due to the small number of studies per subgroup, these findings are hypothesis‐generating and require confirmation in larger datasets.

**FIGURE 7:**
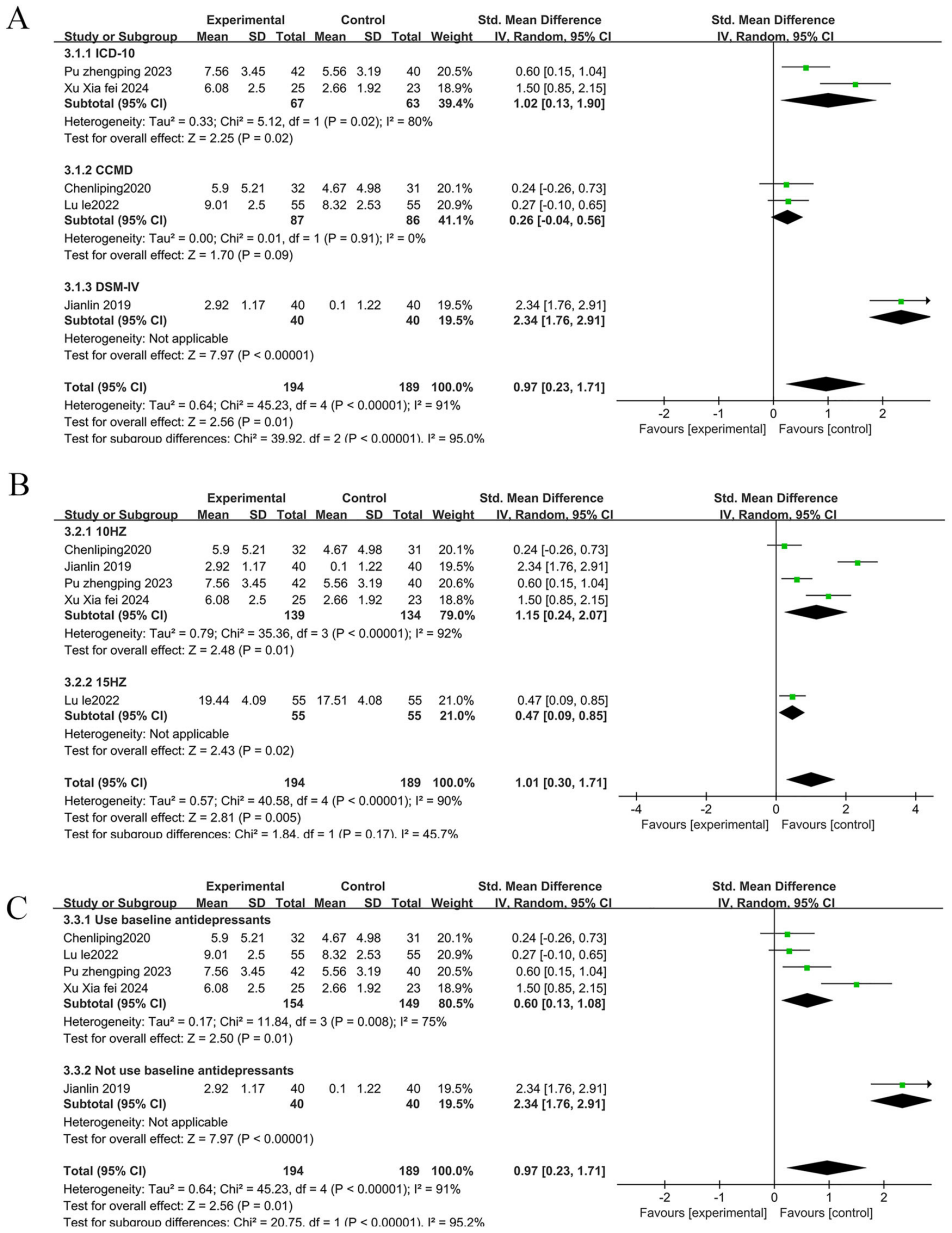
Subgroup analyses. (A) By diagnostic criteria: forest plot comparing the effects across studies using international classification of diseases, 10th revision (ICD‐10), Chinese Classification of Mental Disorders (CCMD), and Diagnostic and Statistical Manual of Mental Disorders, Fourth Edition (DSM‐IV) diagnostic criteria. The pooled effect sizes were 0.90 (95% CI: 0.13, 1.80, *I* ^2^ = 80%) for ICD‐10, 0.26 (95% CI: −0.04, 0.56, *I* ^2^ = 0%) for CCMD, and 2.34 (95% CI: 1.76, 2.91) for DSM‐IV (single study). The test for subgroup differences indicated a statistically significant difference (*p* < 0.00001, *I* ^2^ = 95.0%), suggesting that diagnostic criteria significantly moderate the overall effect; (B) By stimulation frequency: forest plot comparing studies utilizing 10 Hz and 15 Hz. The pooled effect size was 1.15 (95% CI: 0.24, 2.07, *I* ^2^ = 92%) for the 10 Hz subgroup and 0.47 (95% CI: 0.09, 0.85) for the 15 Hz subgroup. The test for subgroup differences showed no significant difference (*p* = 0.17, *I* ^2^ = 45.7%); (C) By baseline antidepressant use: forest plot comparing studies with and without baseline antidepressant use. The pooled effect size was 0.60 (95% CI: 0.13, 1.08, *I* ^2^ = 75%) for the baseline antidepressant subgroup and 2.34 (95% CI: 1.76, 2.91) for the no baseline antidepressant subgroup (single study). The test for subgroup differences indicated a highly significant difference (*p* < 0.00001, *I* ^2^ = 95.2%).

The MDD and substance‐included subgroups showed significant improvements, while the PSD subgroup did not reach statistical significance (Figure S1). SL also exhibited high heterogeneity; however, as only two studies were included, the source of heterogeneity could not be reliably identified through subgroup analysis. Therefore, a stepwise exclusion method was adopted to examine possible influences of outliers or methodological differences. Details are available in Table S1.

### 3.5 Certainty of Evidence (GRADE)

The GRADE assessment is presented in Table 3. The overall certainty of evidence ranged from very low to low. For sleep quality (PSQI), the certainty was rated as very low, downgraded for very serious inconsistency (*I* ^2^ = 91%), serious indirectness (heterogeneous populations and sham protocols), imprecision (CIs crossing the minimal important difference), and suspected publication bias. For depressive symptoms (HAMD‐17), the certainty was low; downgrading was due to risk of bias (blinding difficulties) and indirectness, while inconsistency was not serious (*I* ^2^ = 0%). For SL and AEs, certainty was also very low, primarily due to very few studies and events, wide CIs, and high heterogeneity for SL. These findings suggest that future high‐quality research is very likely to change the current effect estimates.

**TABLE 3:** GRADE summary of findings certainty of evidence for HF‐rTMS versus sham.

Outcome	No. of studies (participants)	Risk of bias	Inconsistency	Indirectness	Imprecision	Publication bias	Effect estimate (95% CI)	Certainty (GRADE)	Downgrading reasons
**Sleep quality (PSQI total score)**	7 RCTs (*N* = 709)	Serious	Very serious	Serious	Serious	Suspected	SMD 0.97 (0.23–1.71)	**⊕◯◯◯ Very low**	^①, ②, ③, ④, ⑤^
**Depressive symptoms (HAMD‐17)**	7 RCTs (*N* = 709)	Serious	Not serious	Serious	Serious	Suspected	SMD 0.61 (0.38–0.84)	**⊕⊕◯◯ Low**	^①, ③, ④, ⑤^
**Sleep latency (SL)**	2 RCTs (*N* = 145)	Serious	Very serious	Serious	Very serious	Not assessable	SMD 0.50 (–0.17–1.18)	**⊕◯◯◯ Very low**	^①, ②, ③, ⑥^
**Adverse events**	7 RCTs (*N* = 709)	Serious	Not serious	Serious	Very serious	Suspected	RR 1.09 (0.80–1.48)	**⊕◯◯◯ Very low**	^①, ③, ⑤, ⑥^

*Note*: **^①^Risk of bias**: studies had unclear allocation concealment and/or inadequate blinding of participants and personnel, which is inherent to rTMS trials.

**^②^Inconsistency**: substantial statistical heterogeneity (*I* ^2^ = 91% for PSQI; *I* ^2^ = 75% for sleep latency) that could not be fully explained by pre‐specified subgroup analyses.

**^③^Indirectness**: heterogeneous patient populations (e.g., post‐stroke depression, substance‐use disorder, elderly) and variable sham stimulation protocols, limiting generalizability to a well‐defined depression‐insomnia comorbidity.

**^④^Imprecision**: confidence intervals crossed the minimal important difference (MID) threshold or the null value, and/or total sample sizes were below the optimal information size (OIS).

**^⑤^Publication bias**: most included studies originated from a single country (China) with predominantly positive findings; formal funnel plot or Egger's test was not performed due to the small number of studies (< 10 per outcome).

**^⑥^Very serious imprecision**: very few studies/events and extremely wide confidence intervals, precluding any reliable conclusion of benefit or harm.

## 4 Discussion

This meta‐analysis synthesized the evidence from existing RCTs. The results showed that left DLPFC‐targeted HF‐rTMS (≥ 5 Hz) significantly improved the core depressive symptoms (HAMD), effectively enhanced sleep quality (PSQI), and appeared to reduce SL in subjects with comorbid depression and insomnia. These findings provide preliminary evidence that HF‑rTMS may confer benefits as a nonpharmacological therapeutic approach for this comorbid population. However, several methodological considerations warrant careful interpretation, and we conducted a formal GRADE assessment to contextualize the strength of the evidence. First, included studies encompassed heterogeneous clinical subtypes (e.g., PSD, addiction/discontinuation populations, and elderly populations), which likely contributed to the substantial heterogeneity in sleep outcomes (*I* ^2^ = 91% for PSQI). Second, blinding of participants and personnel is inherently challenging in rTMS trials, and the quality and adequacy of sham procedures varied considerably across studies (e.g., tilted coils vs. sham coils with auditory mimics), potentially introducing performance bias. Third, our GRADE evaluation rated the overall certainty of evidence as very low for sleep quality, SL, and AEs, and low for depressive symptoms, primarily due to serious inconsistency, indirectness, imprecision, and suspected publication bias. Therefore, although the pooled estimates show positive signals, these findings should be interpreted as exploratory and hypothesis‑generating rather than conclusive. Future large‑scale, sham‑controlled trials with standardized protocols and objective sleep measures are needed to upgrade the evidence certainty and confirm the clinical utility of HF‑rTMS in this population.

The comorbidity mechanism between depression and insomnia stems from functional dysregulation at key nodes within the prefrontal–limbic system. Abnormal activity in the medial dorsal prefrontal cortex—the “dorsal hub”—enhances pathological coupling between the default mode network and affective network. This disrupts the downregulation of default mode network activity and the emotional decoupling required for sleep (Phillips et al. 2003; Sheline et al. 2010). Simultaneously, the tonic inhibitory effect of the anterior cingulate cortex on the amygdala is attenuated, leading to overactivation of the stress hormone axis and persistently elevated sympathetic nervous system excitability. These are key downstream effects underlying prolonged SL and fragmented sleep (Nutt et al. 2008; Pezawas et al. 2005). Chronic deprivation of slow‐wave sleep further diminishes the cognitive control resources mediated by the DLPFC, resulting in sustained high levels of emotional arousal even during rest periods (Fox et al. 2012; Nutt et al. 2008). HF‐rTMS applied to the left DLPFC can enhance its functional connectivity with the anterior cingulate cortex and restore the regulatory weight of the cognitive control network over the limbic system. This resolves the aforementioned abnormal coupling at the functional connectivity level (Kan et al. 2023; Tik et al. 2017). Ultimately, such intervention helps liberate the default mode network from persistent emotional interference, thereby simultaneously improving both sleep continuity and emotional stability (He et al. 2025; Watkins and Roberts 2020). Established evidence suggests that left DLPFC‐rTMS enhances prefrontal‐amygdala functional connectivity (Tik et al. 2017). Hypothetically, this may help uncouple the abnormally heightened connectivity between the default mode and affective networks observed in depression‐insomnia comorbidity (Sheline et al. 2010). However, direct evidence linking rTMS‐induced network changes to sleep improvement is currently lacking.

The orexin/hypocretin system plays a central role in wakefulness and hyperarousal regulation (Mogavero et al. 2023). Whether HF‐rTMS modulates this pathway directly or indirectly remains unknown, but this represents a promising avenue for future mechanistic studies combining rTMS with cerebrospinal fluid or neuroimaging biomarkers.

Based on large initial clinical samples, multicenter designs, and favorable safety and tolerability data, the 10‐Hz left DLPFC rTMS protocol has received FDA clearance for MDD (O'Reardon et al. 2007) and is also recommended as Level A evidence in clinical practice guidelines (Lefaucheur et al. 2020). While some studies suggest that frequencies above 10 Hz may be effective, this could be related to individual brainwave patterns (Fitzgerald et al. 2013; He et al. 2025; Zrenner et al. 2018). Therefore, most researchers prefer to use 10 Hz stimulation in the left dorsolateral prefrontal region. It is necessary to optimize personalized parameters in future research.

In current clinical practice, commonly used first‐line antidepressants encompass selective serotonin reuptake inhibitors, serotonin and norepinephrine reuptake inhibitors, and norepinephrine‐specific serotonergic antidepressants (Cipriani et al. 2018), which demonstrate sedative and insomnia‐relieving effects (Marwaha et al. 2023). Additionally, there are many types of antidepressants (Cipriani et al. 2018). The specific antidepressant chosen by a doctor is based on the patient's physical condition; not all patients use the same drug, which is also the reason for heterogeneity.

Regarding the question of antidepressant‐independent versus antidepressant‐dependent sleep effects, a central question is whether the observed sleep improvement represents a direct effect of rTMS on sleep‐wake circuits or a secondary consequence of overall mood amelioration. Our study‐level data cannot disentangle these mechanisms, as both HAMD and PSQI improved in parallel. While some preclinical evidence suggests that prefrontal rTMS may modulate thalamocortical oscillations and orexinergic pathways independently of mood effects, this hypothesis requires confirmation using polysomnography and carefully designed mediation analyses in future trials. Clinically, regardless of the mechanism, the co‐improvement of sleep and mood is therapeutically desirable.

Based on a limited number of heterogeneous RCTs, left‐DLPFC HF‐rTMS appears to be a promising adjunctive therapy for improving depressive symptoms and sleep quality in patients with depression and insomnia symptoms. However, high heterogeneity, the small evidence base, and the lack of objective sleep measures warrant large‐scale, well‐designed trials to confirm these findings.

## 5 Limitations

The limitations are as follows. First, although subgroup analyses identified stimulation frequency and baseline medication use as the primary sources of PSQI heterogeneity, they did not produce personalized rTMS protocols specifying optimal targets and parameters, nor did they identify a unified set of safe and effective antidepressants. Second, the inclusion of only seven RCTs resulted in a relatively small sample size, possibly yielding insufficient statistical evidence. Because of the small number of eligible trials, funnel plot analysis and Egger's test would have had insufficient power and were therefore not performed; however, the high non‐response rate may have introduced selection bias, and the findings should be regarded as exploratory. Third, while clinical subtypes, age groups, and treatment duration may represent possible sources of heterogeneity, further subgroup analyses were precluded by the small number of eligible studies. Future research is necessary to explore these factors. Larger trials are needed to provide more robust evidence for clinical practice. Moreover, TMS parameters should be optimized to offer more valuable clinical references for the combined treatment of depression and insomnia.

Publication bias could not be formally assessed using funnel plots or Egger's tests due to the small number of included studies (< 10 per outcome). Nevertheless, the predominance of studies from a single country (China) and the overrepresentation of positive results may suggest publication bias. Readers should therefore interpret the pooled estimates as exploratory rather than confirmatory.

## 6 Conclusions

Evidence of very‑low to low certainty suggests that left DLPFC‑targeted HF‑rTMS may improve sleep quality and depressive symptoms in patients with depression and insomnia. However, given serious methodological limitations and the low certainty of evidence, these findings remain preliminary and require confirmation through large‑scale, well‑designed, sham‑controlled trials using standardized protocols and objective sleep measures.

## Author Contributions

**Wenqing Yang**: Writing – original draft, Writing – review and editing, conceptualization. **Songbin Chen**: methodology. **Xiaotong Li**: funding acquisition. **Junhui Chen**: resources. **Li Li**: supervision. **Jin Yu**: formal analysis, investigation. All authors provided feedback on earlier versions of the manuscript and approved the final version.

## Funding

The authors have nothing to report.

## Ethics Statement

The authors have nothing to report.

## Consent

The authors have nothing to report.

## Conflicts of Interest

The authors report no conflicts of interest.

## Supporting information




**Supporting File 1**: brb371808‐sup‐0001‐SuppMat.docx
**Table S1**: Leave‐one‐out sensitivity analysis for the primary outcome. Data are shown as pooled effect size (SMD, 95% CI) after sequentially excluding each individual study. *I* ^2^ values > 50% indicate substantial heterogeneity; “not applicable” indicates that heterogeneity could not be calculated with only one study remaining. Abbreviations: CCMD‐3, Chinese classification of mental disorders‐3; CI, confidence interval; DSM‐IV, Diagnostic and Statistical Manual of Mental Disorders‐Fourth Edition; HAMD‐17, 17‐item Hamilton Depression Rating Scale; ICD‐10, international classification of diseases‐10; PSQI, Pittsburgh Sleep Quality Index; SL, sleep latency; SMD, standardized mean difference.
**Table S2: Literature search results**. Search terms and Boolean combinations used in PubMed, Embase, Cochrane Library, Web of Science, and Chinese databases (CNKI and Wanfang). Results represent the number of records retrieved by each search set. MeSH = Medical Subject Headings; Emtree = Embase Subject Headings; ti, ab, kw = title, abstract, keyword; SU = subject terms (for Chinese databases).
**Table S3: PRISMA‐2020 checklist**. Items are reported according to the PRISMA 2020 statement (Page et al., 2021). The table indicates the section/topic, item number, checklist item description, and the corresponding page number/line number or section/paragraph where each item is addressed in the manuscript. A separate PRISMA 2020 for Abstracts checklist is also provided for the abstract.
**Table S4: Date extraction table**: Data include study characteristics, participant demographics, diagnostic criteria, intervention parameters, and outcome measures (PSQI, HAMD‐17, sleep latency, adverse events, etc.). Values are shown as mean ± SD where available. T = active rTMS; C = sham/control; NA = not available.
**Table S5: Corresponding author's previous publication record**: The table lists the title, journal, year, and author contribution role for each publication. Journals are indexed in SCI (with impact factor and/or Quartile) or Chinese core databases (PKU Core). Corresponding authorship is indicated where applicable.
**Figure S1: Forest plot of subgroup analysis by population: MDD (1 study)**: significant improvement (*p* = 0.008); **PSD (2 studies)**: non‐significant (*p* = 0.18), with high heterogeneity (*I* ^2^ = 89%)**; substance‐included (1 study)**: highly significant improvement (*p* < 0.00001).
**Figure S2: Forest plot of sensitivity analysis excluding high‐risk studies (3 studies)**. Pooled SMD = 1.02 (95% CI: ‐0.07 to 2.11, *p* = 0.07); heterogeneity: *I* ^2^ = 94%, *p* < 0.00001.

## Data Availability

The datasets generated and analyzed during the current research are accessible from the corresponding author upon reasonable request.
