# Supplementary material for: Efficacy of High‐Frequency Transcranial Magnetic Stimulation Therapy for Depression With Insomnia Symptoms: A Systematic Review and Meta‐Analysis Based on Randomized Controlled Trials
Source: Brain Behav. 2026 Oct 27;16(11):e71808. doi: 10.1002/brb3.71808 (PMC13714347; doi:10.1002/brb3.71808)
Supplement: Supplementary file 1 [file BRB3-16-e71808-s001.docx]

**Table S1.** Leave-one-out sensitivity analysis for the primary outcome

| **Outcome** | **Study Excluded** | **Pooled Effect Size (95% CI)** | **I² (%)** |
| --- | --- | --- | --- |
| PSQI | Main analysis (all 5) | 0.97[0.23, 1.71] | 91 |
|  | Excluding Chenliping2020 | 1.15[0.26, 2.05] | 92 |
|  | Excluding  Jianlin2019 | 0.60[0.13, 1.08] | 75 |
|  | Excluding  Lule2022 | 1.15[0.24, 2.07] | 92 |
|  | Excluding  Puzhengping2023 | 1.07[0.09, 2.05] | 93 |
|  | Excluding  Xuxiafei2024 | 0.84[0.00, 1.69] | 92 |
| HAMD-17 | Main analysis (all 4) | 0.61[0.38, 0.84] | 0 |
|  | Excluding Chenliping2020 | 0.58 [0.33, 0.84] | 0 |
|  | Excluding Lule2022 | 0.70[0.41, 0.99] | 0 |
|  | Excluding Puyzhengping2023 | 0.58[0.31,0.85] | 0 |
|  | Excluding Xuxiafei2024 | 0.60[0.35,0.85] | 0 |
| SL | Main analysis (all 2) | 0.50 [-0.17, 1.18] | 75 |
|  | Excluding Chenliping2020 | 0.84 [0.39, 1.29] | Not applicable |
|  | Excluding Puzhengping2023 | 0.15 [-0.34, 0.65] | Not applicable |
| ICD-10 | Main analysis (all 2) | 1.02 [0.13, 1.90] | 80 |
|  | Excluding Puzhengping2023 | 1.50 [0.85, 2.15] | Not applicable |
|  | Excluding Xuxiafei2024 | 0.60 [0.15, 1.04] | Not applicable |
| CCMD-3 | Main analysis (all 2) | 0.26[-0.04, 0.56] | 0 |
|  | Excluding Chenliping2020 | 0.27 [-0.10, 0.65] | Not applicable |
|  | Excluding Lule2022 | 0.24 [-0.26, 0.73] | Not applicable |
| DSM-IV | Main analysis (all 1)Jianlin2019 | 2.34 [1.76, 2.91] | Not applicable |

Notes:

Effect sizes are presented as standard mean differences (SMD) with 95% confidence intervals (CI).

I² statistic quantifies heterogeneity; values > 50% indicate substantial heterogeneity.

**Table S2:** Literature Search Results

PUBMED

| Set | Search term | Search type | Results |
| --- | --- | --- | --- |
| #1 | **depressive disorder[MeSH Terms]** | advanced | 128996 |
| #2 | “ Depressive Disorders“ OR ”Disorder, Depressive“ OR ”Disorders, Depressive“ OR ”Neurosis, Depressive“ OR ”Depressive Neuroses“ OR ”Depressive Neurosis“ OR ”Neuroses, Depressive“ OR ”Depression, Endogenous“ OR ”Depressions, Endogenous“ OR ”Endogenous Depression“ OR ”Endogenous Depressions“ OR ”Melancholia“ OR ”Melancholias“ OR ”Unipolar Depression“ OR ”Depression, Unipolar“ OR ”Depressions, Unipolar“ OR ”Unipolar Depressions“ OR ”Depressive Syndrome“ OR ”Depressive Syndromes“ OR ”Syndrome, Depressive“ OR ”Syndromes, Depressive“ OR ”Depression, Neurotic“ OR ”Depressions, Neurotic“ OR ”Neurotic Depression“ OR ”Neurotic Depressions“ OR ”Depressive Symptoms“ OR ”Depressive Symptom“ OR ”Symptom, Depressive“ OR ”Emotional Depression“ OR ”Depression, Emotional ” |  | 98947 |
| #3 | **Sleep Initiation and Maintenance Disorders[MeSH Terms]** |  | 20246 |
| #4 | “ Disorder, Sleep Wake“ OR ”Disorders, Sleep Wake“ OR ”Sleep Wake Disorder“ OR ”Wake Disorder, Sleep“ OR ”Wake Disorders, Sleep“ OR ”Sleep Disorders“ OR ”Disorder, Sleep“ OR ”Disorders, Sleep“ OR ”Sleep Disorder“ OR ”Long Sleeper Syndrome“ OR ”Long Sleeper Syndromes“ OR ”Sleeper Syndrome, Long“ OR ”Sleeper Syndromes, Long“ OR ”Syndrome, Long Sleeper“ OR ”Syndromes, Long Sleeper“ OR ”Short Sleeper Syndrome“ OR ”Short Sleeper Syndromes“ OR ”Sleeper Syndrome, Short“ OR ”Sleeper Syndromes, Short“ OR ”Syndrome, Short Sleeper“ OR ”Syndromes, Short Sleeper“ OR ”Short Sleep Phenotype“ OR ”Phenotype, Short Sleep“ OR ”Phenotypes, Short Sleep“ OR ”Short Sleep Phenotypes“ OR ”Sleep Phenotypes, Short“ OR ”Sleep-Related Neurogenic Tachypnea“ OR ”Neurogenic Tachypnea, Sleep-Related“ OR ”Neurogenic Tachypneas, Sleep-Related“ OR ”Sleep Related Neurogenic Tachypnea“ OR ”Sleep-Related Neurogenic Tachypneas“ OR ”Tachypnea, Sleep-Related Neurogenic“ OR ”Tachypneas, Sleep-Related Neurogenic“ OR ”Subwakefullness Syndrome“ OR ”Subwakefullness Syndromes“ OR ”Syndromes, Subwakefullness“ OR ”Syndrome, Subwakefullness“ OR ”Circadian Rhythm Sleep Disorder“ OR ”Sleep-Wake Schedule Disorders“ OR ”Sleep Wake Schedule Disorders“ OR ”Sleep-Wake Cycle Disorders“ OR ”Sleep Wake Cycle Disorders“ OR ”Circadian Rhythm Sleep Disorders“ OR ”Disturbed Nyctohemeral Rhythm“ OR ”Disturbed Nyctohemeral Rhythms“ OR ”Nyctohemeral Rhythm, Disturbed“ OR ”Nyctohemeral Rhythms, Disturbed“ OR ”Sleep-Wake Cycle Disorder“ OR ”Sleep Wake Cycle Disorder“ OR ”Sleep-Wake Schedule Disorder“ OR ”Sleep Wake Schedule Disorder“ OR ”Shift-Work Sleep Disorder“ OR ”Shift Work Sleep Disorder“ OR ”Sleep Disorder, Shift-Work“ OR ”Shift-Work Sleep Disorders“ OR ”Sleep Disorder, Shift Work“ OR ”Sleep Disorders, Shift-Work“ OR ”Advanced Sleep Phase Syndrome“ OR ”Delayed Sleep Phase Syndrome“ OR ”Delayed Sleep-Phase Syndrome“ OR ”Delayed Sleep-Phase Syndromes“ OR ”Non-24 Hour Sleep-Wake Disorder“ OR ”Non 24 Hour Sleep Wake Disorder“ OR ”Sleep-Wake Disorder, Non-24 Hour“ OR ”Sleep Wake Disorder, Non 24 Hour“ OR ”Nonorganic Sleep Wake Cycle Disorders“ OR ”Nonorganic Sleep Wake Cycle Disorder“ OR ”DIMS (Disorders of Initiating and Maintaining Sleep)“ OR ”Disorders of Initiating and Maintaining Sleep“ OR ”Sleeplessness“ OR ”Insomnia Disorder“ OR ”Insomnia Disorders“ OR ”Insomnia“ OR ”Insomnias“ OR ”Chronic Insomnia“ OR ”Insomnia, Chronic“ OR ”Early Awakening“ OR ”Awakening, Early“ OR ”Nonorganic Insomnia“ OR ”Insomnia, Nonorganic“ OR ”Primary Insomnia“ OR ”Insomnia, Primary“ OR ”Psychophysiological Insomnia“ OR ”Insomnia, Psychophysiological“ OR ”Rebound Insomnia“ OR ”Insomnia, Rebound“ OR ”Secondary Insomnia“ OR ”Insomnia, Secondary“ OR ”Sleep Initiation Dysfunction“ OR ”Dysfunction, Sleep Initiation“ OR ”Dysfunctions, Sleep Initiation“ OR ”Sleep Initiation Dysfunctions“ OR ”Transient Insomnia“ OR ”Insomnia, Transient ” |  | 55625 |
| #5 | Transcranial magnetic stimulation[MeSH Terms] |  | 16967 |
| #6 | “magnetic stimulation, transcranial'  ”OR ”stimulation, transcranial magnetic“ OR ”transcranial magnetic stimulation' “ OR ”TMS“ OR ”rTMS” |  | 29702 |
| #7 | （#1 OR #2）AND (#3 OR #4 ) AND (#5 OR #6) |  | 108 |
|  |  |  |  |
|  |  |  |  |
|  |  |  |  |

Embase

| Set | Search term | Search type | Results |
| --- | --- | --- | --- |
| #1 | **'central depression'**:ti,ab,kw OR **'clinical depression'**:ti,ab,kw OR **'depressive disease'**:ti,ab,kw OR **'depressive disorder'**:ti,ab,kw OR **'depressive episode'**:ti,ab,kw OR **'depressive illness'**:ti,ab,kw OR **'depressive personality disorder'**:ti,ab,kw OR **'depressive state'**:ti,ab,kw OR **'depressive symptom'**:ti,ab,kw OR **'depressive syndrome'**:ti,ab,kw OR **'depressivity'**:ti,ab,kw OR **'mental depression'**:ti,ab,kw OR **'parental depression'**:ti,ab,kw OR **'depression'**:ti,ab,kw | Search | 768492 |
| #2 | **'depression'**/exp | Emtree | 723618 |
| #3 | **'insomnia'/exp** | Emtree | 103083 |
| #4 | ((((**'agrypnia'**:ti,ab,kw OR **'disorder of sleep initiation'**:ti,ab,kw) AND **maintenance**:ti,ab,kw OR **'disorders of sleep initiation'**:ti,ab,kw) AND **maintenance**:ti,ab,kw OR **'hyposomnia'**:ti,ab,kw OR **'hyposomnias'**:ti,ab,kw OR **'insomnia disorder'**:ti,ab,kw OR **'insomnia disorders'**:ti,ab,kw OR **'insomnias'**:ti,ab,kw OR **'sleep initiation'**:ti,ab,kw) AND **'maintenance disorder'**:ti,ab,kw OR **'sleep initiation'**:ti,ab,kw) AND **'maintenance disorders'**:ti,ab,kw OR **'sleep initiation/maintenance disorder'**:ti,ab,kw OR **'sleeplessness'**:ti,ab,kw OR **'insomnia'**:ti,ab,kw | Search | 62768 |
| #5 | **'transcranial magnetic stimulation'**/exp | Emtree | 40120 |
| #6 | #3 OR #4**'magnetic stimulation, transcranial'**:ti,ab,kw OR **'stimulation, transcranial magnetic'**:ti,ab,kw OR **'transcranial magnetic stimulation'**:ti,ab,kw | Search | 45490 |
| #7 | #1 OR #2 |  | 1016705 |
| #8 | #3 OR #4 |  | 113728 |
| #9 | #5 OR #6 |  | 52435 |
| #10 | #7 AND #8 AND #9 |  | 465 |

COchrane

| Set | Search term | Search type | Results |
| --- | --- | --- | --- |
| #1 | MeSH descriptor: [Depressive Disorder] explode all trees | MeSH | 17037 |
| #2 | **(central depression' OR 'clinical depression' OR 'depressive disease' OR 'depressive disorder' OR 'depressive episode' OR 'depressive illness' OR 'depressive personality disorder' OR 'depressive state' OR 'depressive symptom' OR 'depressive syndrome' OR 'depressivity' OR 'mental depression' OR 'parental depression' OR 'depression'):ti,ab,kw** | Search | 61956 |
| #3 | **MeSH descriptor: [Sleep Initiation and Maintenance Disorders] explode all trees** | MeSH | 3981 |
| #4 | ‘Sleeplessness;' OR ' Insomnia Disorders; ' OR 'Insomnia Disorder;' OR ' Insomnias; Insomnia;' OR ' Secondary Insomnia; ' OR 'Insomnia, Secondary; ' OR 'Transient Insomnia; ' OR 'Insomnia, Transient; ' OR 'Insomnia, Rebound; ' OR 'Rebound Insomnia; ' OR 'Insomnia, Psychophysiological; ' OR 'Psychophysiological Insomnia; ' OR 'Disorders of Initiating and Maintaining Sleep; ' OR 'DIMS (Disorders of Initiating and Maintaining Sleep);' OR ' Early Awakening; Awakening, Early; ' OR 'Insomnia, Primary;' OR ' Primary Insomnia;' OR ' Chronic Insomnia; ' OR 'Insomnia, Chronic;' OR ' Insomnia, Nonorganic; ' OR 'Nonorganic Insomnia;' OR ' Dysfunctions, Sleep Initiation; ' OR 'Sleep Initiation Dysfunctions; ' OR 'Sleep Initiation Dysfunction; ' OR 'Dysfunction, Sleep Initiation' | Search | 16659 |
| #5 | MeSH descriptor: [Transcranial Magnetic Stimulation] explode all trees | MeSH | 2783 |
| #6 | ‘Stimulation, Transcranial Magnetic; ' OR 'Magnetic Stimulations, Transcranial; ' OR 'Magnetic Stimulation, Transcranial; ' OR 'Transcranial Magnetic Stimulations; ' OR 'Stimulations, Transcranial Magnetic; ' OR 'Transcranial Magnetic Stimulation, Single Pulse; ' OR 'Transcranial Magnetic Stimulation, Repetitive; ' OR 'Transcranial Magnetic Stimulation, Paired Pulse;' OR 'TMS;' OR 'rTMS' | Search | 10920 |
| #7 | **(#1 OR #2) AND(#3 OR #4) AND(#5 OR #6)** | Search | 113 |

Wed of Science

| Set | Search term | Search type | Results |
| --- | --- | --- | --- |
| #1 | “ Depressive Disorders“ OR ”Disorder, Depressive“ OR ”Disorders, Depressive“ OR ”Neurosis, Depressive“ OR ”Depressive Neuroses“ OR ”Depressive Neurosis“ OR ”Neuroses, Depressive“ OR ”Depression, Endogenous“ OR ”Depressions, Endogenous“ OR ”Endogenous Depression“ OR ”Endogenous Depressions“ OR ”Melancholia“ OR ”Melancholias“ OR ”Unipolar Depression“ OR ”Depression, Unipolar“ OR ”Depressions, Unipolar“ OR ”Unipolar Depressions“ OR ”Depressive Syndrome“ OR ”Depressive Syndromes“ OR ”Syndrome, Depressive“ OR ”Syndromes, Depressive“ OR ”Depression, Neurotic“ OR ”Depressions, Neurotic“ OR ”Neurotic Depression“ OR ”Neurotic Depressions“ OR ”Depressive Symptoms“ OR ”Depressive Symptom“ OR ”Symptom, Depressive“ OR ”Emotional Depression“ OR ”Depression, Emotional ” | Topic | 182001 |
| #2 | “DIMS (Disorders of Initiating and Maintaining Sleep)“ OR ”Disorders of Initiating and Maintaining Sleep“ OR ”Sleeplessness“ OR ”Insomnia Disorder“ OR ”Insomnia Disorders“ OR ”Insomnia“ OR ”Insomnias“ OR ”Chronic Insomnia“ OR ”Insomnia, Chronic“ OR ”Early Awakening“ OR ”Awakening, Early“ OR ”Nonorganic Insomnia“ OR ”Insomnia, Nonorganic“ OR ”Primary Insomnia“ OR ”Insomnia, Primary“ OR ”Psychophysiological Insomnia“ OR ”Insomnia, Psychophysiological“ OR ”Rebound Insomnia“ OR ”Insomnia, Rebound“ OR ”Secondary Insomnia“ OR ”Insomnia, Secondary“ OR ”Sleep Initiation Dysfunction“ OR ”Dysfunction, Sleep Initiation“ OR ”Dysfunctions, Sleep Initiation“ OR ”Sleep Initiation Dysfunctions“ OR ”Transient Insomnia“ OR ”Insomnia, Transient ” | Search | 87695 |
| #3 | “magnetic stimulation, transcranial'  ”OR ”stimulation, transcranial magnetic“ OR ”transcranial magnetic stimulation' “ OR ”TMS“ OR ”rTMS” | Search | 64224 |
| #4 | #1 AND #2 AND # 3 |  | 27 |

| 数据库 | 检索式 | 数量 |
| --- | --- | --- |
| 知网 | (SU='抑郁' OR SU='抑郁症' OR SU='焦虑抑郁' OR SU='抑郁症状' OR SU='抑郁情绪') AND (SU='失眠' OR SU='失眠症' OR SU='失眠症状' OR SU='失眠多梦' OR SU='失眠患者') AND (SU='重复经颅磁刺激' OR SU='重复经颅磁刺激治疗' OR SU='rTMS') | 66 |
| 万方 | ((题名或关键词=(抑郁 OR 抑郁 OR 焦虑抑郁 OR 抑郁症状 OR 抑郁情绪)) AND 题名或关键词=(失眠 OR 失眠症 OR 失眠症状 OR 失眠多梦 OR 失眠患者 OR 失眠症患者)) AND 题名或关键词=(重复经颅磁刺激 OR 重复经颅磁刺激治疗 OR 重复经颅磁刺激技术 OR rTMS) | 10 |

**Table S3:** PRISMA-2020-Checklist

**PRISMA 2020 Checklist**

| **Section/topic** | **Item No** | **Checklist item** | **Reported on Page Number/Line Number** | **Reported on Section/Paragraph** |
| --- | --- | --- | --- | --- |
| **TITLE** | | | | |
| Title | 1 | Identify the report as a systematic review. | 1 | 1 |
| **ABSTRACT** | | | | |
| Abstract | 2 | See the PRISMA 2020 for Abstracts checklist (Table 2). | 167-168 | 3.1 |
| **INTRODUCTION** | | | | |
| Rationale | 3 | Describe the rationale for the review in the context of existing knowledge. | 45-59 | 1 |
| Objectives | 4 | Provide an explicit statement of the objective(s) or question(s) the review addresses. | 68-73 | 1 |
| **METHODS** | | | | |
| Eligibility criteria | 5 | Specify the inclusion and exclusion criteria for the review and how studies were grouped for the syntheses. | 87-103 | 2..3 |
| Information sources | 6 | Specify all databases, registers, websites, organisations, reference lists and other sources searched or consulted to identify studies. Specify the date when each source was last searched or consulted. | 76-86,167 | 2.1-2.2,3.1 |
| Search strategy | 7 | Present the full search strategies for all databases, registers and websites, including any filters and limits used. | 82-86 | 3.1 |
| Selection process | 8 | Specify the methods used to decide whether a study met the inclusion criteria of the review, including how many reviewers screened each record and each report retrieved, whether they worked independently, and if applicable, details of automation tools used in the process. | 120-159 | 2.5-2.7 |
| Data collection process | 9 | Specify the methods used to collect data from reports, including how many reviewers collected data from each report, whether they worked independently, any processes for obtaining or confirming data from study investigators, and if applicable, details of automation tools used in the process. | 120-159 | 2.5-2.7 |
| Data items | 10a | List and define all outcomes for which data were sought. Specify whether all results that were compatible with each outcome domain in each study were sought (e.g. for all measures, time points, analyses), and if not, the methods used to decide which results to collect. | 105-119 | 2.4 |
|  | 10b | List and define all other variables for which data were sought (e.g. participant and intervention characteristics, funding sources). Describe any assumptions made about any missing or unclear information. | 105-119 | 2.4 |

| Study risk of bias assessment | 11 | Specify the methods used to assess risk of bias in the included studies, including details of the tool(s) used, how many reviewers assessed each study and whether they worked independently, and if applicable, details of automation tools used in the process. | 120-158 | 2..5-2.7 |
| --- | --- | --- | --- | --- |
| Effect measures | 12 | Specify for each outcome the effect measure(s) (e.g. risk ratio, mean difference) used in the synthesis or presentation of results. | 140-159 | 2.7 |
| Synthesis methods | 13a | Describe the processes used to decide which studies were eligible for each synthesis. | 162-167 | 3.1 |
|  | 13b | Describe any methods required to prepare the data for presentation or synthesis, such as handling of missing summary statistics, or data conversions. | 217-223 | 3.4 |
|  | 13c | Describe any methods used to tabulate or visually display results of individual studies and syntheses. | 162-172 | 3.1 |
|  | 13d | Describe any methods used to synthesize results and provide a rationale for the choice(s). If meta-analysis was performed, describe the model(s), method(s) to identify the presence and extent of statistical heterogeneity, and software package(s) used. | 133-158 | 2.6-2.7 |
|  | 13e | Describe any methods used to explore possible causes of heterogeneity among study results. | 182-223 | 3.3.1-3.4 |
|  | 13f | Describe any sensitivity analyses conducted to assess robustness of the synthesized results. | 221-223 | 3.4 |
| Reporting bias assessment | 14 | Describe any methods used to assess risk of bias due to missing results in a synthesis (arising from reporting biases). | 132-139 | 2.6 |
| Certainty assessment | 15 | Describe any methods used to assess certainty (or confidence) in the body of evidence for an outcome. | 140-159 | 2.7 |
| **RESULTS** | | | | |
| Study selection | 16a | Describe the results of the search and selection process, from the number of records identified in the search to the number of studies included in the review, ideally using a flow diagram. | 161-172 | 3.1 |
|  | 16b | Cite studies that met many but not all inclusion criteria (‘near-misses’) and explain why they were excluded. | 163-172 | 3.1 |
| Study characteristics | 17 | Cite each included study and present its characteristics. | 163-172 | 3.1 |
| Risk of bias in studies | 18 | Present assessments of risk of bias for each included study. | 174-179 | 3.2 |
| Results of individual studies | 19 | For all outcomes, present, for each study: (a) summary statistics for each group (where appropriate) and (b) an effect estimate and its precision (e.g. confidence/credible interval), ideally using structured tables or plots. | 181-223 | 3.3 |

| Results of syntheses | 20a | For each synthesis, briefly summarise the characteristics and risk of bias among contributing studies. | 180-223 | 3.3 |
| --- | --- | --- | --- | --- |
|  | 20b | Present results of all statistical syntheses conducted. If meta-analysis was done, present for each the summary estimate and its precision (e.g. confidence/credible interval) and measures of statistical heterogeneity. If comparing groups, describe the direction of the effect. | 180-220 | 3.3 |
|  | 20c | Present results of all investigations of possible causes of heterogeneity among study results. | 180-220 | 3.3 |
|  | 20d | Present results of all sensitivity analyses conducted to assess the robustness of the synthesized results. | 180-220 | 3.3 |
| Reporting biases | 21 | Present assessments of risk of bias due to missing results (arising from reporting biases) for each synthesis assessed. | 180-220 | 3.3 |
| Certainty of evidence | 22 | Present assessments of certainty (or confidence) in the body of evidence for each outcome assessed. | 180-220 | 3.3 |
| **DISCUSSION** | | | | |
| Discussion | 23a | Provide a general interpretation of the results in the context of other evidence. | 226-299 | 4 |
|  | 23b | Discuss any limitations of the evidence included in the review. | 226-299 | 4 |
|  | 23c | Discuss any limitations of the review processes used. | 226-299 | 4 |
|  | 23d | Discuss implications of the results for practice, policy, and future research. | 226-299 | 4 |
| **OTHER INFORMATION** | | | | |
| Registration and protocol | 24a | Provide registration information for the review, including register name and registration number, or state that the review was not registered. | 79-80 | 2.1 |
|  | 24b | Indicate where the review protocol can be accessed, or state that a protocol was not prepared. | 79-80 | 2.1 |
|  | 24c | Describe and explain any amendments to information provided at registration or in the protocol. | NA | NA |
| Support | 25 | Describe sources of financial or non-financial support for the review, and the role of the funders or sponsors in the review. | NA | NA |
| Competing interests | 26 | Declare any competing interests of review authors. | NA | NA |
| Availability of data, code and other materials | 27 | Report which of the following are publicly available and where they can be found: template data collection forms; data extracted from included studies; data used for all analyses; analytic code; any other materials used in the review. | Tables 1-3 | Tables 1-3 |

**Table S3 PRISMA 2020 for Abstracts checklist**

| **Section/topic** | **Item No** | **Checklist item** | **Reported on Page Number/Line Number** | **Reported on Section/Paragraph** |
| --- | --- | --- | --- | --- |
| **TITLE** | | | | |
| Title | 1 | Identify the report as a systematic review. | 1 | 1 |
| **BACKGROUND** | | | | |
| Objectives | 2 | Provide an explicit statement of the main objective(s) or question(s) the review addresses. | 68-73 | 1 |
| **METHODS** | | | | |
| Eligibility criteria | 3 | Specify the inclusion and exclusion criteria for the review. | 87-103 | 2.3 |
| Information sources | 4 | Specify the information sources (e.g. databases, registers) used to identify studies and the date when each was last searched. | 76-86,167 | 2.1-2.2,3.1 |
| Risk of bias | 5 | Specify the methods used to assess risk of bias in the included studies. | 132-139 | 2.6 |
| Synthesis of results | 6 | Specify the methods used to present and synthesize results. | 141-158 | 2.7 |
| **RESULTS** | | | | |
| Included studies | 7 | Give the total number of included studies and participants and summarise relevant characteristics of studies. | 162-172 | 3.1 |
| Synthesis of results | 8 | Present results for main outcomes, preferably indicating the number of included studies and participants for each. If meta-analysis was done, report the summary estimate and confidence/credible interval. If comparing groups, indicate the direction of the effect (i.e. which group is favoured). | 182-223 | 3.3.1-3.4 |
| **DISCUSSION** | | | | |
| Limitations of evidence | 9 | Provide a brief summary of the limitations of the evidence included in the review (e.g. study risk of bias, inconsistency and imprecision). | 302-318 | 5 |
| Interpretation | 10 | Provide a general interpretation of the results and important implications. | 302-318 | 5 |
| **OTHER** | | | | |
| Funding | 11 | Specify the primary source of funding for the review. | NA | NA |
| Registration | 12 | Provide the register name and registration number. | 79-80 | 2.1 |

Please leave this space alone as it will be supplemented by the editorial office when needed.

**Table S4: Date extraction table**

|  | **Basic research information** | | | **Characteristics of the research object** | | | | | | | | **Intervention measures** | | **Outcome** | | | | | |
| --- | --- | --- | --- | --- | --- | --- | --- | --- | --- | --- | --- | --- | --- | --- | --- | --- | --- | --- | --- |
| **number** | **Author** | **Year** | **Country** | **Sample size** | **Sample size (experimental group/control group)** | **Follow-up time** | **Fale/Female** | **Age,years** | **Diagnostic criteria** | **Complications** | **Baseline medication** | **Stimulus parameter** | **Equipment and Solutions** | **Total PSQI score changes (baseline/post-treatment** | **Total score changes of HAMD-17 (baseline/post-treatment** | **Clinical remission rate** | **Sleep latency** | **Adverse reactions** | Quality of life （ SF-36） |
| 1 | PuZhengPing | 2023 | China | 82 | T=42 C =40 | 8week | T group: 42 males, 19 females, 31 Sham group: 40 males, 22 females, 28 | T :35.24 ± 5.12 C：33.97 ± 4.74 | (1) Mild or moderate depressive episodes, or recurrent depression, with the current episode being mild or moderate; (2) Non-organic insomnia; According to the International Classification of Diseases (10th Edition) ICD-10, it meets the inclusion criteria of this experiment. (3)17 points ≤ (HAMD) score <23 points; (PSQI)≥7 points; | NA | Agomelatine 25–75 mg/day × 8 weeks | HF-rTMS:（L-DLPFC）10（Hz），120%MT、25 reps × 8 sets, 26s rest, 5×/wk, 4 wks (20 sessions) Sham rTMS with identical parameters and coil placement, no magnetic field | YIRUIDE (Wuhan) & Agomelatine | T0:14.62±3.84; T4 7.06±2.87; T8 4.38±1.24); C0 14.24±3.67; C4 8.68±2.11; C8 6.72±1.53) | T0 22.82±6.39; T4 8.57±3.25; T8 4.43±1.13); C0 21.45±6.56; C4 11.13±3.74; C8 6.39±1.27 | (4,72）= 23.66 | T0 61.32±10.55; T8 24.47±5.71 C0 58.28±11.17; C8 29.52±3.36 | rTMS AEs: headache (2), dizziness (4), scalp paresthesia (2), miosis (1). Agomelatine AEs: dizziness (3), nausea (4), fatigue (2), liver function abnormality (1) | NA |
| 2 | William J | 2010 | USA | 48 | T =18 C=7 | 1-3months | Female 29, male 19 | T:46.7±15.3 C :41.9±14.1 | DSM-IV MDD, SCID-confirmed. HAMD-24 total ≥18 and item-1 ≥3 at two screenings | NA | Patients continued antidepressants during study. | Tgroup：L-DLPFC,100%MT，5HZ。2000 stimuli/day: 40 stimuli × 50 sets, 8s/set, 22s interval, 2 weeks. Sham coil: identical appearance/sound to active Magstim 8-shaped coil, but output attenuated by 90% via magnetic shielding. | Magstim Super Rapid stimulator (Magstim, Dyfed, UK) with high-power figure-8 coil (mean diameter 7 cm) | NA | T0:28.2±6.0 T4:18.2±9.8 T12:16.3±11.5 C0:27.7± 3.5 C4：26.0±9.2 C12:25.8±7.4 | NA | NA | Active (n=30): dizzy 14, scalp discomfort 10, eye pain 3, neck sore 2, facial twitch 1, lightheaded 5, nausea 6, fatigue 6, insomnia 3, inattention 1. Sham (n=11): dizzy 6, scalp discomfort 3, lightheaded 2, fatigue 2, insomnia 3, inattention 1. | NA |
| 3 | Peter B | 2013 | USA | 301 | T =155 C=146 | 2-6week | NA | T :47.9±11.0 C:48.7±10.6 | DSM-IV non-psychotic unipolar MDD (SCID-IV-confirmed). Moderate-to-severe baseline symptoms and treatment resistance (ATHF) in current episode. | NA | Treatment for depression; limited hypnotics/anxiolytics permitted. Mean acute-phase doses: active 8.7 vs sham 6.7 | Left DLPFC (5 cm anterior to MT), 120% MT, 10 Hz, 4s ON, 26s OFF, 3000 pulses/session, 5×/wk, 6 wks. | NeuroStar TMS System (model 2100, Neuronetics, Malvern, PA, USA). | NA | T0HF-TMS:30.7±3.9 C0shamTMS:30.6± 4.3 | NA | NA | Active group: insomnia 24.2%, hypersomnia 0.6%; Sham: insomnia 25.3%, hypersomnia 1.3%; Rescue anxiolytics/hypnotics: active 12.2%, sham 18.2%. | NA |
| 4 | Jianlin | 2019 | China | 105 | T =40 C =40 | 6week | Male | T：33.95±7.13 C：34.5±8.54 | Drug dependence (DSM-IV, psychiatrist interview). Inclusion: (1) withdrawal completed: drug-free, methadone-free ≥6mo, morphine urine (-), naloxone (-); (2) no acute symptoms, normal labs; (3) no psychiatric/personality disorders; (4) no HIV/syphilis. | NA |  | L-DLPFC 10Hz rTMS, 100% RMT, 5s ON/10s OFF, 10 min, 40 trains, 2000 pulses. Localized via EEG 10/20 system. | Figure-8 coil (CCY-I TMS device, YIRUIDE, Wuhan, China). | T0:10.95± 1.34 T6:8.03±0.83  C0:12.78±1.25  C6:12.68±1.19 | NA | NA | NA | Mild dizziness/scalp pain: intervention 3, sham 1, control 0. | NA |
| 5 | XuXiaFei | 2024 | China | 74 | T=25 C =23 | 6week | T males 14 (56%); Control: males 11 (47.8%) | T:60.62±11.01 C:59.6±10.54 | Cerebrovascular disease (4th Conference criteria), CT/MRI: hemorrhage/infarction, first-episode, 6-24mo, conscious, independent scales.  PSD (2016 Chinese consensus), HAMD-17 ≥7.   PSQI ≥8 (past mo).  Cooperated; consent signed. ICD-10: depression/insomnia. | Stroke duration: 6–24 months. | Ischemic: aspirin (Bayer 0.1g) + atorvastatin (Pfizer 20mg). Hemorrhagic: risk factor management (HTN, DM) + targeted therapy. | T: 10Hz, 90%, 4s/26s, 75 trains, 3000 pulses, 2250s, 5/wk ×10. Sham: coil vertical (edge contact), auditory clicks, same protocol | CCY-I 8-coil (YIRUIDE, Wuhan) + paroxetine 25-50mg/d; clonazepam 1-3mg PRN (max 5 doses). | T0:16.96± 2.59 T2:10.88± 2.42 T6:9.44± 2.00 C0:16.83±1.87 C2:14.17±1.97 C6:13.57±3.36 | T0:28.72 ± 7.87 T2:11.40 ± 4.21 T6:9.28± 3.77 C0:29.87±5.82 C2:16.70±4.24 C6:11.83±2.92 | NA | NA | Mild transient headache/dizziness/nausea (n=6), did not impact study | NA |
| 6 | Chen LiPing | 2020 | China | 63 | T=32 C=31 | 4week | T:32 (23M/9F); C: 31 (21M/10F) | T:64.06±6.82 C:65.16±9.18 | ① Depression/insomnia per CCMD-3; ② HAMD-17 ≥8; ③ PSQI ≥8. | Post-stroke >2 months. | Vasodilators, neurotrophic agents, and escitalopram oxalate | T: 10Hz/90%MT, L-DLPFC (tangential), 5s×20 trains, 1min ITI, 1/d×10d. Sham: vertical coil (edge contact), clicks, same protocol. | Magstim rTMS with figure-8 coil, combined with vasodilators, neurotrophic agents, and escitalopram oxalate. | T0:12.06 ±5.85 T4:6.16± 4.17 C0:11.52±5.51 C4:6.85±4.20 | T0:20.25± 2.24 T4:8.35± 4.58 C0:20.28±2.13 C4:11.06±3.81 | NA | T0: 56.63±24.09  T4:43.63±13.09  C0:55.40±23.90 C4:45.63±15.09 | 8 subjects had transient mild headache/dizziness/nausea, did not affect experiment. | NA |
| 7 | LuLe | 2022 | China | 110 | T=55 C=55 | 12week | T: 22M/23F; C: 27M/28F | T:33.85±8.16 C:35.04±9.22 | ① CCMD-2 depression; ② PSQI >5; ③ insomnia >3 months. | NA | Agomelatine tablets (25 mg, Hansoh Pharma, Jiangsu, China) | T: bilateral DLPFC & M1, coil tangential to scalp; 15Hz (5s ON/10s OFF, 10min) then 1Hz (5s ON/10s OFF, 10min), 80-85% RMT, 1/d, 5/wk ×12wk. Sham: coil perpendicular (90°), same parameters otherwise. | rTMS device (MagPro R30, Tonica Elektronik A/S) with figure-8 coil, combined with agomelatine tablets (25mg, Hansoh Pharma, Jiangsu). | T0:15.11± 2.76 T12:6.10± 2.14 C0:15.29±2.84 C12:6.97±2.03 | T0:28.23± 4.41 T12:8.61± 0.73 C0:28.05±4.57 C12:10.54±1.26 | Overall response rate: active 49%, control 4%.1 | NA | T: headache/dizziness 9, somnolence 6, elevated transaminases 7, GI discomfort 7. C: headache/dizziness 12, somnolence 4, elevated transaminases 8, GI discomfort 10. | NA |

Data include study characteristics, participant demographics, diagnostic criteria, intervention parameters, and outcome measures (PSQI, HAMD‑17, sleep latency, adverse events, etc.). Values are shown as mean ± SD where available. T = active rTMS; C = sham/control; NA = not available.

**Figure S1: Forest plot of subgroup analysis by population: MDD (1 study):** significant improvement (p = 0.008); **PSD (2 studies):** non-significant (p = 0.18), with high heterogeneity (I² = 89%)**; substance-included (1 study):** highly significant improvement (p < 0.00001).

**Figure S2: Forest plot of sensitivity analysis excluding high-risk studies (3 studies).** Pooled SMD = 1.02 (95% CI: -0.07 to 2.11, p = 0.07); heterogeneity: I² = 94%, p < 0.00001.
